# Theoretical and modelling investigation of pendant groups effect on quantum interference for single-molecule junctions†

**DOI:** 10.1039/d4ra01463d

**Published:** 2024-05-07

**Authors:** Oday A. Al-Owaedi

**Affiliations:** a Department of Laser Physics, College of Science for Women, University of Babylon Hilla 51001 Iraq oday.alowaedi@uobabylon.edu.iq; b Al-Zahrawi University College Karbala, Najaf-Karbala Street 56001 Iraq

## Abstract

Quantum interference (QI) is one of the most important phenomena that affects the charge transport through single molecules. The effect of a constructive and destructive quantum interference on electronic, thermoelectric and spectroscopic properties of oligo(phenyleneethynylene) based-molecular junctions has been investigated using a combination of density functional theory (DFT) methods, tight binding (Hückel) modelling (TBHM) and quantum transport theory (QTT). Molecules with carbonyl, diphenyl, ethane and ethynylferrocene substituents show a destructive quantum interference (DQI), which enhances thermoelectric properties of these molecules making them promising materials for thermoelectric applications.

## Abstract 2

Impacts of meta-connectivity and pendant groups are robust parameters controlling the destructive quantum interference (DQI) and improve thermoelectric properties of meta-OPE molecules making them suitable materials for thermoelectric applications.

## Graphical abstract

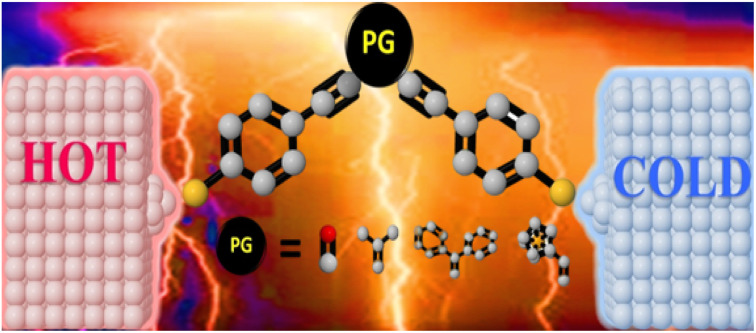

## Introduction

Room-temperature quantum interference (QI) has become a powerful strategy to examine the properties of single molecular junctions and their applications.^1–5^ Numerous theoretical and experimental studies have explored QI and the quantum circuit rules using oligo(phenyleneethynylene) (OPE) molecules, with a focus on the connectivity type (*para*, *ortho* and *meta*).^6–11^ Venkataraman *et al.*^12^ have found that QI is vital to all molecular-scale electron transport, and innovated a QI map which allows one to easily identify individual QI effects. Additionally, they mentioned that comparing *meta*- and *para*-benzene further illustrates the connection between QI and molecular orbital coefficients. Lambert *et al.*^13^ innovated a new rule which captures a minimal description of connectivity-driven charge transport and provides a useful starting point for chemists to design appropriate molecules for molecular electronics with desired functions. The magic ratio rule (MRR) predicts conductance ratios, which are solely determined by QI within the core of polycyclic aromatic hydrocarbons (PAHs). The manifestations of QI and related quantum circuit rules for materials discovery are direct consequences of the key concepts of weak coupling, locality, connectivity, mid-gap transport and phase coherence in single-molecule junctions. The impact of various terminal and central parts on the properties of OPE molecules has been studied widely.^14–19^ Exploring the role of the central part of *para*- or *meta*-substituted oligo(phenylene-ethynylene) molecules in establishing the quantum interference has become a goal of a wide range of research studies.^20–23^ There are various functional structures that could be employed as a central part. For example, Yonghai Li *et al.*^24^ reported an experimental and theoretical investigation of the redox-state impact of pendant diimide units on the charge transport through core-substituted naphthalenediimide (NDI) single-molecule junctions. Their study suggests that integration of a pendant redox unit with strong coupling to a molecular backbone enables the tuning of charge transport through single-molecule devices by controlling their redox states. In this context, this work explores the influence of carbonyl pendant group, which distinguished by a polar covalent bond between carbon and oxygen atoms, and the electronegativity of this substituent may play a crucial role in establishing and determining QI.^25,26^ The aromaticity and rotation aspects of diphenyl group could be another source of QI.^27,28^ In addition, the rotary phase property of the ethane group could play an important role in examine the quantum interference.^29,30^ Furthermore, the π donor–acceptor interactions which characterized the ethynylferrocene group making this substituent a promising candidate to probe the transport characteristics of OPE-based molecular junctions. Herein, the electronic, thermoelectric and spectroscopic properties of *para* and *meta*-substituted oligo(phenylene-ethynylene) molecules with phenyl, carbonyl, diphenyl, ethane and ethynylferrocene pendant groups at the central part of molecule have been investigated using a combination of density functional theory (DFT) methods,^31,32^ a tight binding (Hückel) modelling (TBHM)^33^ and quantum transport theory (QTT).^34–43^

## Computational methods

The initial optimization of gas phase molecules, isosurfaces and spectroscopic calculations were carried out at the B3LYP level of theory^44^ with 6-31G** basis set^45,46^ using density functional theory (DFT) and time-dependent (TD-DFT)^47^ respectively. Geometrical optimization of all gold|molecule|gold configurations under investigation in this work was carried out by the implementation of DFT^31,32^ in the SIESTA^31^ code, as shown in Fig. S2 (see ESI†). The generalized gradient approximation (GGA) of the exchange and correlation functional is used with a double-*ζ* polarized (DZP) basis set, a real-space grid defined with an equivalent energy cut-off of 250 Ry. The geometry optimization for each structure is performed to the forces smaller than 20 meV Å^−1^. The mean-field Hamiltonian obtained from the converged DFT calculations was combined with GOLLUM^35^ code. The quantum transport theory (QTT)^34–43^ implemented in GOLLUM have been used to calculate the electronic and thermoelectric properties of all molecular junctions.

## Results and discussion

**Fig. 1:**
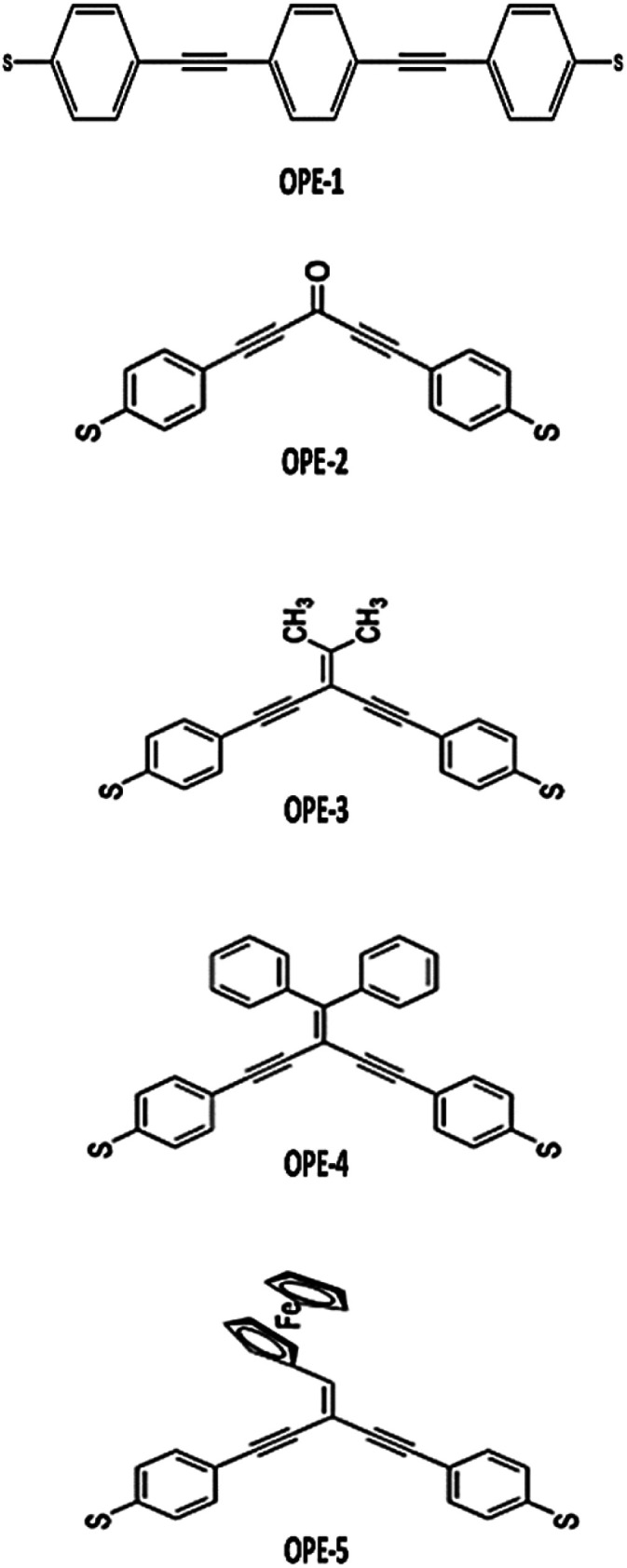
Schematic illustration of OPE molecules.

A family of five *para*- and *meta*-substituted oligo(phenylene-ethynylene) molecules were chosen for study here. In the quest to explore the connectivity type effect and pendant groups on the properties of molecules. The molecules under investigation have been selected with *para* and *meta* connections and four different pendant groups at the central part, as shown in Fig. 1.

**Fig. 2:**
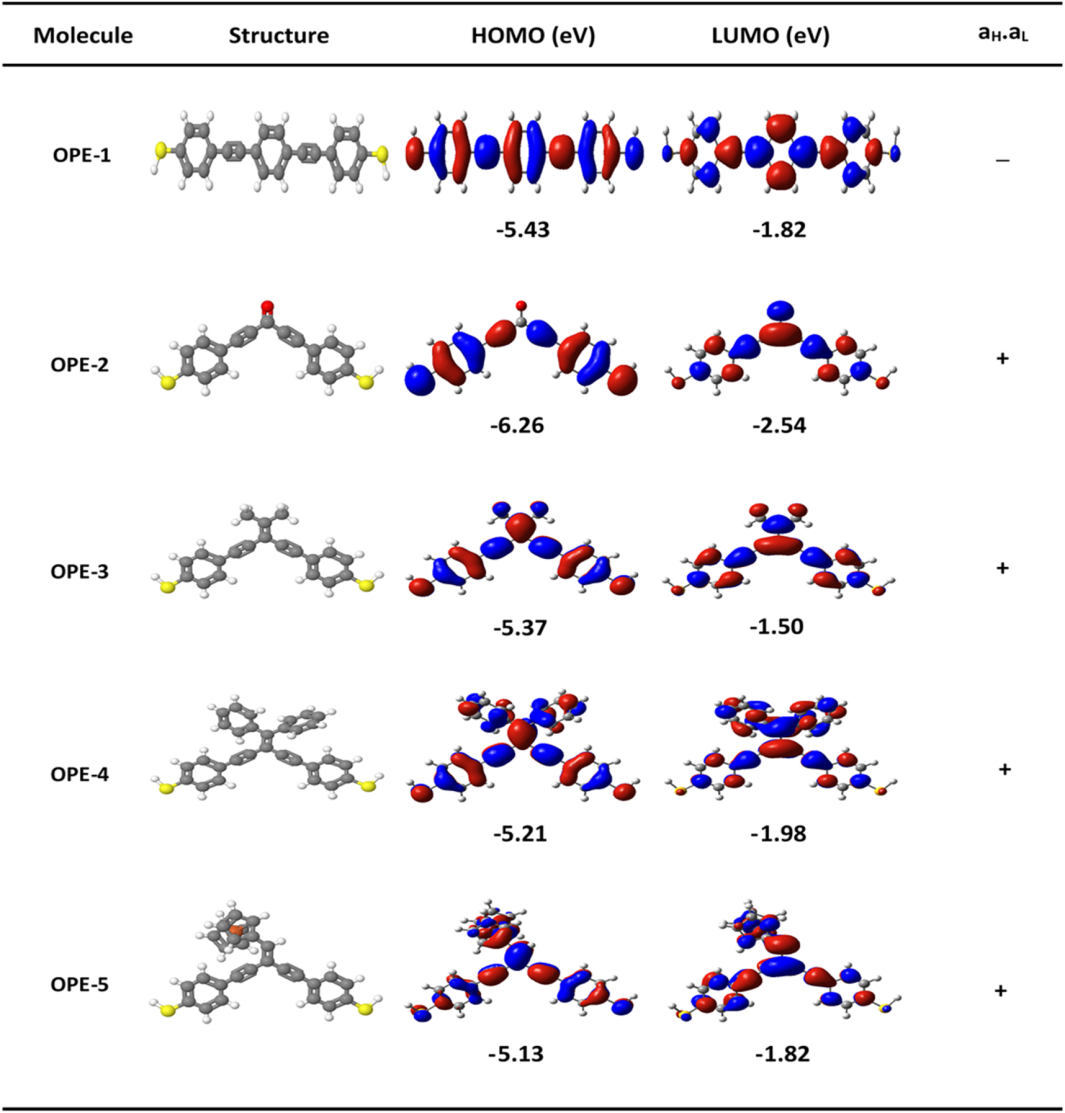
The optimized geometry of all molecules in a gas phase (structure), HOMOs and LUMOs (isosurfaces ±0.02 (e bohr^−3^)^1/2^), blue part is a positive sign, red part is a negative sign. *a*_H_*a*_L_ is the multiplication of the HOMO and LUMO amplitudes. As an example, HOMO and LUMO for OPE-5 molecule possess the same sign, then the multiplication of molecular orbitals amplitudes (*a*_H_*a*_L_) is a positive sign and the molecule exhibits DQI.

Fig. 1 shows that the first molecule (OPE-1) consists of three *para*-connected phenyl rings. While the *meta*-substituted oligo(phenylene-ethynylene) with carbonyl group as a central part is the second molecule (OPE-2). It is well known that the electronegativity of the oxygen atom makes the carbon–oxygen double bonds in this structure are very reactive.^48^ The ethane compound is the central part of the third molecule (OPE-3). Newman *et al.*^49^ have mentioned that the free rotation of CH_3_ fragments around single bonds will produces six unique conformations of the ethane structure (see ESI†). OPE-4 is the fourth molecule with diphenyl as a pendant group. The conformational enantiomorphism is the feature of this group, since one phenyl ring being slightly twisted or canted in relation to the other as a consequence of steric crowding leading to a chiral conformation of the diphenyl group.^50^ The ethynylferrocene group is the central part of the fifth molecule (OPE-5). The ethynylferrocene pendant group consists of two cyclopentadienyl (Cp) rings bound by a central iron atom, which may rotate about the Cp–Fe–Cp axis. In the gas phase the ground state is found to be the staggered configuration, while in other phases it corresponds to the eclipsed configuration.^51^ The orbitals distribution and the electronic structure of molecules were investigated and plots for optimized structures, the highest occupied and lowest unoccupied molecular orbitals (HOMOs and LUMOs, respectively) are given in Fig. 2. HOMOs of all molecules display a familiar pattern of π–π interactions along the molecular backbone. LUMOs are also localized over the molecular backbones and could be described as a π-conjugated system. In addition, Fig. 2 and Table 1 show that the values of HOMO–LUMO gap have been fluctuated and the highest value (3.87 eV) was given by molecule OPE-3, while the lowest value (3.23 eV) was exhibited by molecule OPE-4. These results are consistent with previous studies.^52,53^ Furthermore, the calculated values of HOMO–LUMO gap were ranging from 3.23 to 3.87 eV, which are close to the HOMO–LUMO gap of C_60_ molecule indicating that the OPEs are a promising molecules for materials applications.^54^

**Table 1:** H–L gap is the HOMO–LUMO gap of the molecules in a gas phase; ^*A*^*λ*_Max_ is the maximum absorption wavelength; *A* is the absorption intensity; ^*E*^*λ*_Max_ is the maximum emission wavelength; *E* is the emission intensity; *f*_em_ is emission oscillator strength; SS is the Stokes shift

Molecule	H–L gap (eV)	^*A*^ *λ*_Max_ (nm)	*A* (a.u.)	^*E*^ *λ*_Max_ (nm)	*E* (a.u.)	*f*_em_	SS (nm)
OPE-1	3.61	349.3	2868.4	370.9	91 387.2	2.25	21.6
OPE-2	3.72	349.7	1053.1	370.6	41 285.6	0.96	20.9
OPE-3	3.87	242.8	2688	259	56 700.8	0.93	16.2
OPE-4	3.23	298	1312	320.8	35 227.3	0.74	22.8
OPE-5	3.31	556	7.6	600.8	1033	0.02	44.8

In order to explore the impact of connectivity type and to prove the existence of CQI and DQI in OPE molecules, the current investigation performed an orbital analysis, and demonstrated that DQI is the dominant phenomenon in the transport of all molecular junctions, except the transport through OPE-1 molecular junction, which is dominated by CQI, as shown in Fig. 2. Lambert *et al.*^55^ have reported an orbital symmetry rule. The magic ratio theory^14^ is based on utilising of the exact core Green's function, which is defined by:

1 *g*(*E*) = (*IE* − *H*)^−1^

In the literature, various approximations to *g*(*E*) are discussed, one of which involves the approximation of including only the contributions to *g*(*E*) from the HOMO and LUMO. If the amplitudes of the HOMO on sites a and b are denoted 
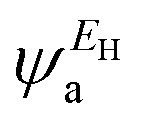
 and 
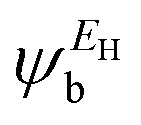
 and the amplitudes of the LUMO are 
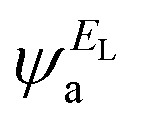
 and 
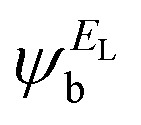
, then if the contributions from all other orbitals are ignored, then, a crude approximation to the Green's function *g*_ab_(*E*) is2
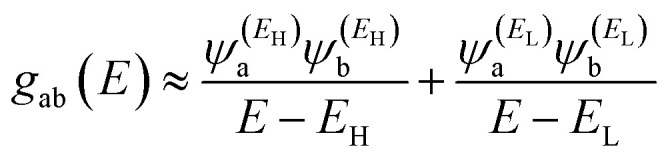
where *E*_H_ and *E*_L_ are the energies of the HOMO and LUMO respectively. If the HOMO product 
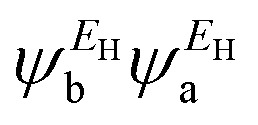
 has the same sign as the LUMO product 
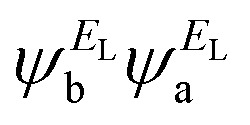
 then the right-hand side of eqn (2) will vanish at some energy *E* in the range *E*_H_ ≤ *E* ≤ *E*_L_. That is for some energy *E* within the HOMO–LUMO gap. In this case, one can say that the HOMO and the LUMO interfere destructively. On the other hand, if the HOMO and LUMO products have opposite signs then the right hand side of eqn (2) will not vanish within the HOMO–LUMO gap and one can say that the HOMO and LUMO interfere constructively within the gap (they could of course interfere destructively at some other energy *E* outside the gap). When the right-hand side of eqn (2) vanishes, the main contribution to *g*_ba_(*E*) comes from all other orbitals, so in general eqn (2) could be a poor approximation. One exception to this occurs when the lattice is bipartite, because the Coulson–Rushbrooke (CR) theorem^56^ tells us that if a and b are both even or both odd, then the orbital products on opposite sides of eqn (3) and (4) have the same sign. Consequently when the HOMO and LUMO interfere destructively, all other pairs of orbitals interfere destructively, leading to the trivial zeros in the magic number table,^14^ for which *g*_ba_(0) = 0.

The equations of Coulson–Rushbrooke (CR) theorem are known as:^56^3
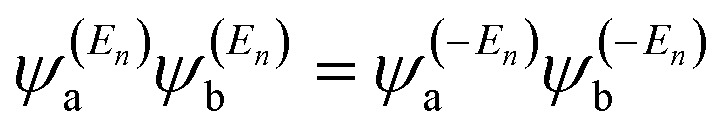
4
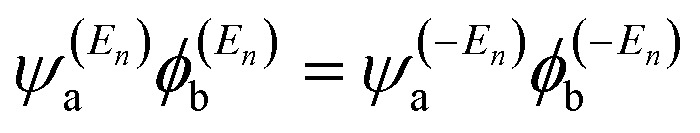
where 
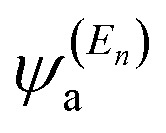
 is a vector of amplitudes on even-numbered sites, and 
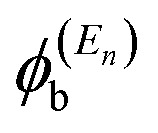
 is a vector of amplitudes on odd-numbered sites. ±*E*_*n*_ are eigenvalues come in ± pairs and the eigenstate belonging to −*E*_*n*_ is related to the eigenstate belonging to *E*_*n*_. Obviously, this exact cancellation is a property of bipartite lattices only, but based on its success for bipartite lattices, one might suppose that eqn (2) is a reasonable approximation, for other lattices. Nevertheless, as pointed out by Yoshizawa *et al.*,^57–60^ since orbitals such as those in Fig. 2 are often available from DFT calculations, it can be helpful to examine the question of whether or not the HOMO and LUMO (or indeed any other pair of orbitals) interfere destructively or constructively, by examining the colours of orbitals. This is simplified by writing eqn (2) in the form5
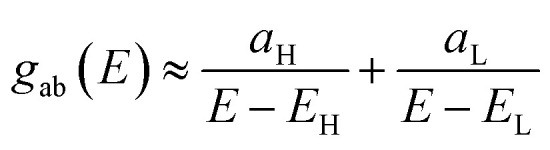
where 
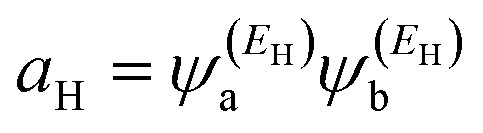
 and 
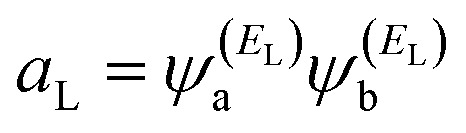
. If the HOMO product *a*_H_ has the same sign as the LUMO product *a*_L_ then the right-hand side of eqn (5) will vanish for some energy *E* in the range *E*_H_ ≤ *E* ≤ *E*_L_. In other words, the HOMO and LUMO will interfere destructively at some energy within the HOMO–LUMO gap. However this does not mean that the exact *g*_ba_(*E*) will vanish. Indeed, if the right hand side of (5) vanishes, then the contributions from all other orbitals become the dominant terms.^61^ It is worth to mention that the colour of HOMO orbitals on sulfur atoms for molecule OPE-2 are not identical, since the colour of HOMO on the sulfur atom at the left side is blue, while the colour on the right side atom is red. In contrast, the colour of LUMO orbitals for both sulfur atoms is red. However, the multiplication of HOMO amplitudes on right and left sides of molecule 
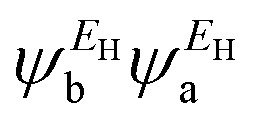
 is a negative sign, which means the sign of *a*_H_ is a negative sign, which is the same sign as the LUMO product 
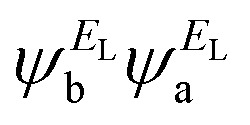
, then the right-hand side of eqn (2) will vanish at some energy *E* in the range *E*_H_ ≤ *E* ≤ *E*_L_. In other words, the production of *a*_H_*a*_L_ is a positive sign, which indicates to a destructive quantum interference. Nevertheless, this is an appealing method of identifying QI effects in molecules and describing their qualitative features.^62^

The distinctive properties of OPE molecules including for example their spectroscopic properties, especially the absorption and emission spectra, have become the subject of the increased interest for many scientific studies.^63–65^ The UV/visible absorption and emission spectra showed asymmetric peaks, since the range of the absorption spectra is extend from 298 to 556 nm, as shown in Table 1, and the emission spectra is ranging from 259 to 600.8 nm. These results could be understood in terms of structural features of these molecules, since OPE-1 molecule possess a high aromaticity, while the high reactivity is the distinguished feature of OPE-2 molecule.^48,49^ Molecules OPE-3 and OPE-4 have staggered and chiral conformations respectively.^50,51^ In contrast, the eclipsed configuration identifies the OPE-5 molecule.^66^

**Fig. 3:**
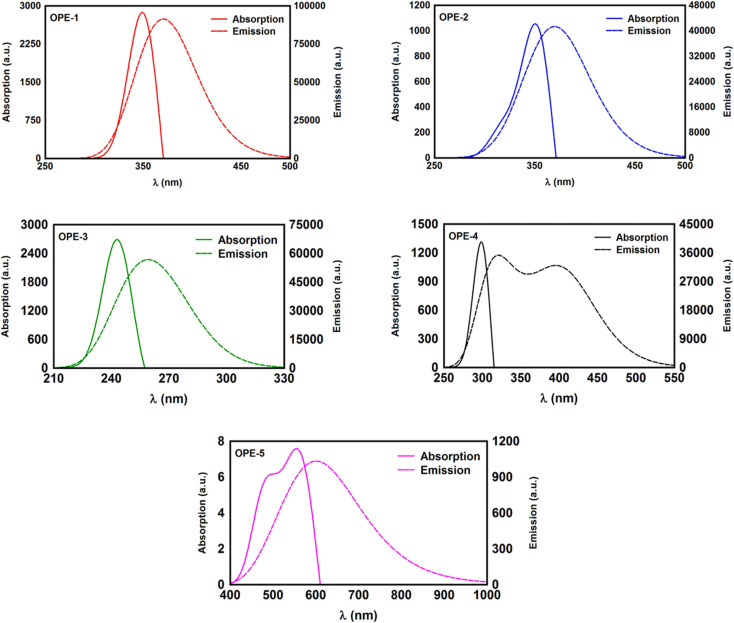
UV/Vis absorption spectra (solid curves) and emission spectra (dashed curves) for all molecules.

In addition, Fig. 3 and Table 1 show that the value of Stokes shift is ranging from 16.2 to 44.8 nm, as shown in Table 1. These results may introduce OPE-5 molecule as a promise candidate for the encryption and medical applications.^67,68^ One of the most important parameters in optoelectronics applications is the emission oscillator strength (*f*_em_).^69^ Theoretically, for a given PL material, *f*_em_ is directly proportional to the emission cross section (*σ*_em_) and it is given by:^70^6
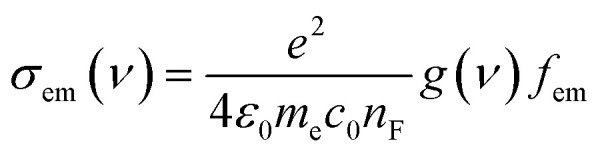
where *e* is the electron charge, *ε*_0_ is the vacuum permittivity, *m*_e_ is the mass of electron, *c*_0_ is the speed of light, *n*_F_ is the refractive index of the gain material, *ν* is the frequency of the corresponding emission, and *g*(*ν*) is the normalized line shape function with ∫*g*(*ν*)d*ν* = 1.

Furthermore, Table 1 shows that three molecules (OPE-1, OPE-2 and OPE-3) have *f*_em_ of 2.25, 0.96 and 0.93 respectively. These results could be ascribed to the highest intensity value of emission spectra of these molecules (91 387.2, 41 285.6 and 56 700.8 a.u. respectively). This means the highest HOMO → LUMO transition rate in these molecules, which might attributed to an increase in their emission cross section (*σ*_em_), which in turn suggesting that OPE-1, OPE-2 and OPE-3 are appropriate molecules for optoelectronics applications.^71^

**Fig. 4:**
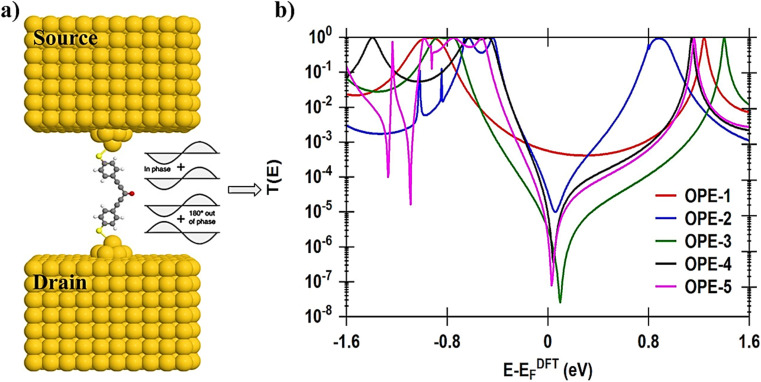
(a) The propagation of de Broglie waves through OPE-2 molecule, which accommodated between source and drain electrodes, as an example; (b) DFT-transmission coefficient *T*(*E*) as a function of electrons energy for all molecular junctions.

Transmission coefficient *T*(*E*) is an essential quantity to characterize the transport properties.^72^ The propagation of de Broglie waves cross source|molecule|drain configuration has been characterized in this work *via* calculating *T*(*E*),^73–77^ as shown in Fig. 4. The transmission coefficient according to Landauer–Büttiker formalism is given by

7 *T*(*E*) = *T*_r_{*Γ*_R_(*E*)*G*^R^(*E*)*Γ*_L_(*E*)*G*^R†^(*E*)}

In this expression,

8 *Γ*_L,R_(*E*) = *i*(*Σ*_L,R_(*E*) − *Σ*^†^_L,R_(*E*))

where *Γ*_L,R_ describes the level broadening due to the coupling between left (L) and right (R) electrodes and the central scattering region, *Σ*_L,R_(*E*) are the retarded self-energies associated with this coupling.

9 *G*^R^ = (*EX* − *H* − *Σ*_L_ − *Σ*_R_)^−1^

where *G*^R^ is the retarded Green's function, *H* is the Hamiltonian and *X* is the overlap matrix. The transport properties is then calculated using the Landauer formula:^78^

10 *G* = *G*_0_∫d*ET*(*E*)(−∂*f*(*E*, *T*)/∂*E*)

where *G*_0_ = 2*e*^2^/*h* is the conductance quantum, *f*(*E*) = (1 + exp((*E* − *E*_F_)/*k*_B_*T*))^−1^ is the Fermi–Dirac distribution function, *T* is the temperature and *k*_B_ = 8.6 × 10^−5^ eV K^−1^ is Boltzmann^'^s constant.^79^

Fig. 4a shows the molecular junction configuration of OPE-2 molecule, as an example (see ESI† for all molecular models). In this work *T*(*E*) has been calculated by attach the optimized molecules with two (111)-directed gold electrodes, which involve two small layers (each one consists of 6-atoms pyramidal gold lead), and seven layers of (111)-oriented bulk gold with each layer consisting of 6 × 6 atoms, and a layer spacing of 0.235 nm to create the molecular junctions. These layers were then further repeated to yield infinitely long gold electrodes which carry the electric current. From these molecular junctions electronic and thermoelectric properties were calculated using GOLLUM code.^35^Fig. 4b shows the transmission coefficient *T*(*E*),^73^ of source|molecule|drain molecular junctions. The signature of constructive quantum interference (CQI) is clear for the first molecule (OPE-1), which leads to the highest *T*(*E*) value (5.67 × 10^−4^), as shown in Table 2. This outcome is ascribed to the *para* connectivity^52,80–85^ between phenyl rings. In contrast, the impact of the *meta* connectivity at the terminal part of molecules along with the effect of pendant groups at the central part resulted to a low *T*(*E*) of the rest of molecules, accompanied with robust antiresonance features at the middle of HOMO–LUMO gap, which is a representation of the destructive quantum interference (DQI).^28–30,86–88^ Feng Jiang *et al.*^89^ have reported experimental results of *meta*-substituted phenylene ethylene type oligomers (*m*-OPE) molecules. They have mentioned that the rotating of the OMe group in a specific conformation (perpendicular to the plane of the molecule) decreases sharply the molecular conductance (log *G*/*G*_0_ = −5.87) through the *m*-OPE moiety. The outcome of their work demonstrate that destructive quantum interference (DQI) can be tuned by changing the position and conformation of methoxy (OMe) substituents at the central phenylene ring. These results are consistent with the results of the current investigation. In addition, the order of *T*(*E*) for the rest molecules is *T*(*E*)_OPE-2_ > *T*(*E*)_OPE-4_ > *T*(*E*)_OPE-3_ > *T*(*E*)_OPE-5_. These results could be interpreted in terms of the molecule length (*l*), which represents the length of the tunnelling barrier, since the order of molecules length is *l*_OPE-2_ < *l*_OPE-4_ < *l*_OPE-3_ < *l*_OPE-5_, as shown in Table 2. The rectangular tunnel barrier model^90^ states that the transmission coefficient *T*(*E*) through a single molecule (barrier) decreases exponentially with the length of the barrier, according to eqn (11).

11 *T*(*E*) ∝ e^−*βl*^

where *T*(*E*) is the transmission coefficient, *β* is the electronic decay constant and *l* is the tunnelling distance. Accordingly, it could be predicted that molecules with the *meta* connectivity and pendant groups might a promising candidates for electronic applications.^71^ Furthermore, the molecule length of OPE-2, OPE-3, OPE-4 and OPE-5 compounds of *ca.* >2 nm is consistent with a dominant contribution of the coherent tunneling transport.^91–94^

**Table 2:** DFT-transmission coefficient *T*(*E*)_DFT_; TBHM-transmission coefficient *T*(*E*)_TBHM_; molecule length (*l* = *S*…*S*); highest occupied molecular orbitals of the molecules in a junction (^J^HOMO); lowest unoccupied molecular orbitals of the molecules in a junction (^J^LUMO); HOMO–LUMO gap (^J^H–L gap) of the molecules in a junction

Molecule	*T*(*E*)_DFT_	*T*(*E*)_TBHM_	*l* (nm)	^J^HOMO (eV)	^J^LUMO (eV)	^J^H–L gap (eV)
OPE-1	5.67 × 10^−4^	—	2.016	0.94	1.24	2.18
OPE-2	3.98 × 10^−5^	3.4 × 10^−5^	1.44	0.63	0.88	1.51
OPE-3	3.6 × 10^−6^	3 × 10^−6^	1.49	0.87	1.4	2.27
OPE-4	1.08 × 10^−5^	1 × 10^−5^	1.47	0.62	1.14	1.76
OPE-5	1.92 × 10^−6^	1.2 × 10^−6^	1.5	0.75	1.16	1.91

**Fig. 5:**
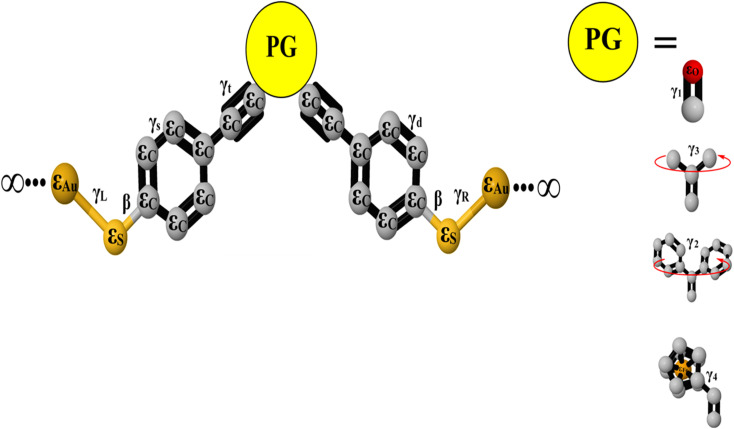
A minimal tight-binding (Hückel) representation of all molecules with different pandent groups. *γ* is the coupling elements, and *ε* is an onsite energy. Grey balls indicates to the onsite energy of carbon atoms (*ε*_C_), light yellow balls refers to the onsite energy of sulfur atoms (*ε*_S_), dark yellow balls is the onsite energy of gold atoms (*ε*_Au_). Red balls indicates to the onsite energy of oxygen atoms (*ε*_O_), Orange balls refers to the onsite energy of iron atoms (*ε*_Fe_) and PG refers to pendant groups. The coupling element between carbon–carbon single bond is *γ*_S_. *γ*_d_ is the coupling element between carbon–carbon double bonds. *γ*_t_ is the coupling element between carbon–carbon triple bonds. *γ*_L_ and *γ*_R_ are the left and right coupling elements between anchor groups and gold electrodes. The coupling element between carbon–oxygen double bonds is *γ*_1_. *γ*_2_ is the coupling element of the dihedral angle between phenyl rings. The coupling element of molecular rotation of CH_3_ fragments is *γ*_3_. *γ*_4_ is the coupling element of twisting angle.

**Fig. 6:**
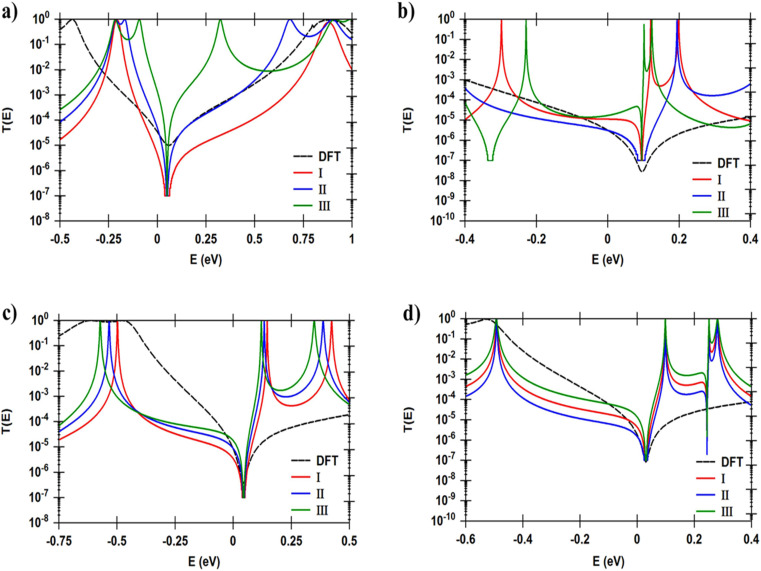
Transmission coefficient as a function of electrons energy for all TBH models presented in Fig. 5; (a) *T*(*E*) of OPE-2 model; (b) *T*(*E*) of OPE-3; (c) *T*(*E*) of OPE-4; (d) *T*(*E*) of OPE-5. The dashed black curve is the DFT-transmission coefficient. For all models, and in all cases (I, II, III), *β* = 1, *γ* = 0.5, *ε*_C_ = 0.1, *ε*_S_ = 2.5, *ε*_Au_ = 0.5, *γ*_S_ = 0.3, *γ*_d_ = 0.5, *γ*_t_ = 0.7, *γ*_L_ = *γ*_R_ = 0.2.

In seeking to confirm and understand the transport behaviour of molecules and the relative effects of different pendant groups, a minimal tight-binding (Hückel) model (TBHM) has been constructed, as shown in Fig. 5. The simplest tight-binding Hamiltonian of the parent is obtained by assigning a site energy *ε* to each diagonal and a nearest neighbor hopping integral *γ* between neighbouring sites, *i.e.*, *H*_*ii*_ = *ε* and *H*_*ij*_ = *γ* if *i*, *j* are nearest neighbours. It is worth to mention, that the neglect of TBHM of the interactions between electrons is considered a major defect, but it remains one of the widely used methods to visualize and understand the electronic properties of molecular junctions.^95^Fig. 5 shows a system connected to two one-dimension electrodes on both sides by weak nearest neighbor couplings *γ*_R_ and *γ*_L_. In fact, one of the drawbacks of this kind of computational methods is the produced energy levels are diminished by a few electron voltages in comparison with the accurate values relative to a vacuum. However, TBHM is consider a powerful computational tool because it is not only takes into account all morphological aspects of molecular junctions, but also assumed that the electron transport is elastic and coherent.^95^ The transmission coefficients produced by TBHM as a function of electrons energy of all models are presented in Fig. 6. The outcomes confirm the existence of the DQI signature, and its impact on the transport behaviour. Fig. 6a shows *T*(*E*) of OPE-2 model with carbonyl group as a central part. All parameters *β* = 1, *γ* = 0.5, *ε*_C_ = 0.1, *ε*_S_ = 2.5, *ε*_Au_ = 0.5, *γ*_S_ = 0.3, *γ*_d_ = 0.5, *γ*_t_ = 0.7, *γ*_L_ = *γ*_R_ = 0.2, are fixed for all cases (I, II, III), except *γ*_1_. For case I (*i.e.* red curve), *γ*_1_ = 2; case II (*i.e.* blue curve), *γ*_1_ = 1, and for case III (*i.e.* green curve), *γ*_1_ = 0.5.

It is well known that the carbonyl pendant group is an important configuration because it causes the chemical reactions of the molecule, and by changing the polar double bond of this structure, one can manipulate the electrical dipole momentum between the negative and positive charges of oxygen and carbon atoms. This process leads to DQI, as shown in Fig. 6a (see ESI† for more details). The results of case II are consistent with DFT results and the outcomes of ref. 96 and 97.

Fig. 6b illustres *T*(*E*) as a function of electrons energy of OPE-4 model in three cases. For case I, *γ*_3_ = 0.4, case II, *γ*_3_ = 0.7, and for case III, *γ*_3_ = 0.3. In the context of chemistry, the methyl fragments of ethane are joined by a carbon–carbon sigma bonds allowing them to rotate about these bonds giving a rise to 6D-conformational isomers, three of them are staggered conformation, while the other three will be in an eclipsed conformation.^98^ This called a rotary phase character determining an internal degree of freedom of molecule, which is the molecular rotation. Therefore, Hückel model describes the molecular rotation by changing the value of *γ*_3_ to obtain different eclipsed conformations (see ESI†). An excellent agreement with DFT has been reached for the case II, as shown in Fig. 6b. A diphenyl compound involves two phenyl rings linked to carbon atom by single carbo–carbon bonds. The aromaticity of diphenyl is distinguished by the π-conjugation property.^99^

The changing of the relative twist angle (*γ*_2_) between the two phenyl rings is expected to decrease the degree of π-conjugation between them, lowering *T*(*E*) and impact the transport behaviour of OPE-3 model, because molecular electron transfer rates scale as the square of the π-overlap^100–103^ (see ESI†). Fig. 6c shows the transmission coefficient of OPE-3 model in three cases. For case I, *γ*_2_ = 0.7; case II, *γ*_2_ = 0.9, and for case III, *γ*_2_ = 1.1. Herein, the visualization of Hückel's model for the twist angle (*γ*_2_) in case II is consistent with DFT calculations. The OPE-5 model contains an ethynylferrocene group, which possess an iron metal accommodated between two rings of cyclopentadienyl (CP), as shown in Fig. 1. These rings could be in a staggered or eclipsed conformation and rotate with low resistance about the CP–Fe–CP axis.^104^ Hückel model depicts the molecular rotation by changing the value of *γ*_4_ to obtain different eclipsed conformations^105^ (see ESI†). Fig. 6d exhibits the transmission coefficient of OPE-5 molecule. Hückel model visualizes the twist angle as *γ*_4_. For case I, *γ*_4_ = 0.3; case II, *γ*_4_ = 0.1, and for case III, *γ*_4_ = 0.5. The matching between TBHM and DFT was excellent for the case II, as shown in Fig. 6d and Table 2.

Exploring the influence of DQI on the transport behaviour, and consequently on the thermoelectric properties is one of the main goals of this work. The slope of *T*(*E*) determines the Seebeck coefficient (*S*), and thus the electronic figure of merit (*Z*_el_*T*). The Seebeck coefficient (*S*), power factor (*P*) and (*Z*_el_*T*), are given by:12
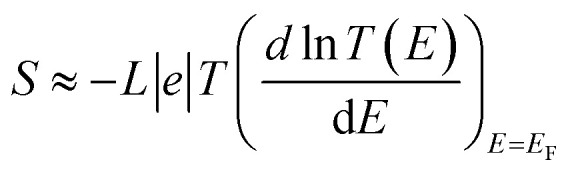
where *L* is the Lorenz number 
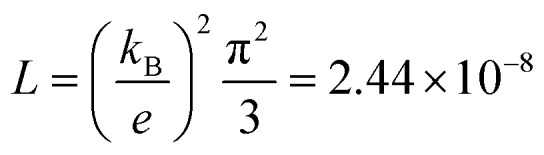
 W Ω K^−2^. In other words, *S* is proportional to the negative of the slope of ln *T*(*E*), evaluated at the Fermi energy. Based on Seebeck coefficient, the power factor was calculated by:

13 *P* = *GS*^2^*T*

where *T* is the temperature *T* = 300 K, *G* is the electrical conductance and *S* is the Seebeck coefficient. The purely electronic figure of merit (*Z*_el_*T*) is given by:^106,107^14
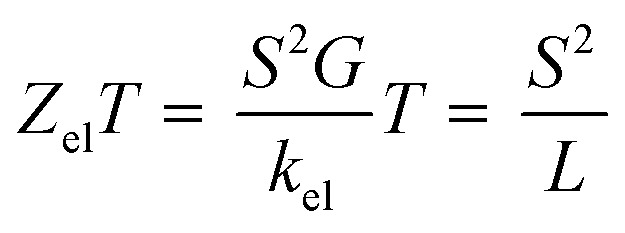
where *k*_el_ is the electron thermal conductance. According to previous studies,^106,107^ the figure of merit in this work was calculated only depending on a purely electronic contribution.

**Fig. 7:**
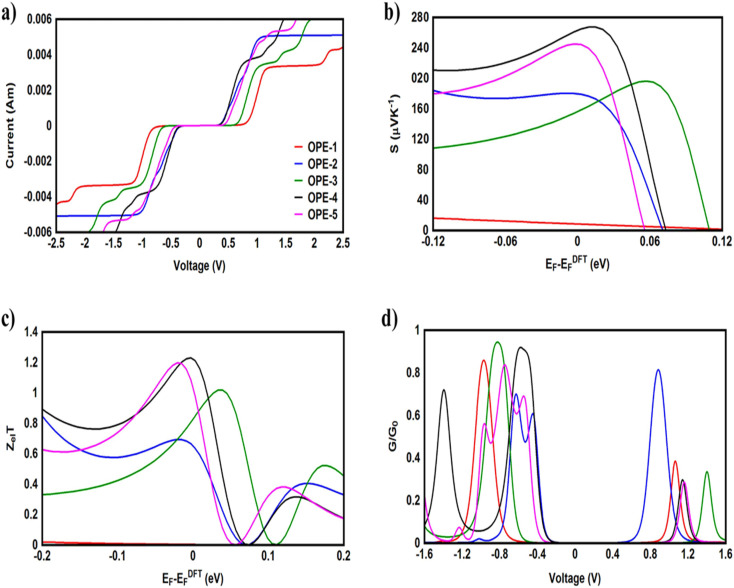
(a) Current–voltage characteristics (*I*–*V*); (b) Seebeck coefficient (*S*); (c) electronic figure of merit (*Z*_el_*T*); (d) electrical conductance (*G*/*G*_0_) as a function of voltage of all molecular junctions.

Fig. 7a and Table 3 present the current–voltage (*I*–*V*) characteristics of all molecular junctions, which are limited to the first and third quadrants of the *I*–*V* plane crossing the origin. Therefore, they are classified as components consume the electric power. Herein the importance of the threshold voltage (*V*_th_) value appears. The values of *V*_th_ for molecules OPE-2, OPE-4 and OPE-5 are 0.36, 0.4 and 0.47 V respectively, which makes these molecules promising candidates for the electronic applications. In addition, *I*–*V* characteristics of all molecular junctions exhibited a quantum staircase behaviour. As the voltage increases, the density of electrons also increases, which leads to an increase in the number of occupied subbands. The dependence conductance in this case is a set of plateaus separated by steps of height 2*e*^2^/*h*: a stepwise change in the conductance of channels occurs each time the Fermi level coincides with one of the subbands. Hence, the quantum staircase behaviour could be attributed to the adiabatic transparency of spin-nondegenerate subbands of these molecules.^62,97^

**Table 3:** Electrical conductance (G/G_0_); Seebeck coefficient (*S*); power factor (*P*); electronic figure of merit (*Z*_el_*T*); threshold voltage (*V*_th_) of all molecular junctions

Molecule	*G*/*G*_0_	*S* (μV K^−1^)	*P* (W K^−1^) × 10^−21^	*Z*_el_ *T*	*V*_th_ (V)
OPE-1	5.35 × 10^−4^	8.7	3103	0.003	0.83
OPE-2	3.46 × 10^−5^	180	97 200	0.66	0.36
OPE-3	3.31 × 10^−6^	154	6640	0.8	0.68
OPE-4	0.97 × 10^−5^	260	56 784	1.22	0.4
OPE-5	1.53 × 10^−6^	116	1883	1.08	0.47

It is well known that the performance of thermoelectric materials is characterized by an efficient conversion of an input heat to the electricity.^95,96,106^ In this context, the enhancement of power factor (*P*) and electronic figure of merit (*Z*_el_*T*) is an important point. Fig. 7b, c and Table 3 show that the highest values of *S* and *Z*_el_*T* (260 μV K^−1^, and 1.22 respectively) have been exhibited by molecule OPE-4. This result agrees with the experimental findings of Hurtado-Gallego *et al.*,^108^ since they reported that the existent of a destructive quantum interference (DQI) in the molecular junction is a consequence of the interaction of the pendant groups (diphenyl group) with the electrodes. Also, the findings of their work proved a positive *S* value indicating HOMO-dominated transport. Consequently, thermoelectric properties of single molecules could be controlled using diphenyl pendant units. On the other hand, molecule OPE-1 presented the lowest values of these parameters (3.13 μV K^−1^, and 0.036 respectively). These results not only demonstrated the important role of DQI for an improvement of the *S* and *Z*_el_*T*, but also established a crucial role of the type of pendant groups^107^ in determining the transport behaviour, *S* and *Z*_el_*T*. In addition, these promising findings of molecule OPE-4 due to the existence of diphenyl rings as pendant groups may intriguing the experimentalists to work out on this kind of compounds, and innovate new pendent groups to improve the properties of thermoelectric materials. Furthermore, the competition between electrical conductance and Seebeck coefficient according to eqn (13) led to the power factor order of *P*_OPE-2_ > *P*_OPE-4_ > *P*_OPE-3_ > *P*_OPE-1_ > *P*_OPE-5_. In light of the aforementioned results, the molecules with pendant groups could be considered promising candidates for thermoelectric applications.

## Conclusions

In conclusions, the effects of connectivity and pendant groups are robust parameters in controlling the quantum interference (QI) and improve thermoelectric properties of *meta*-substituted oligo(phenylene-ethynylene) (*meta*-OPE) molecules, making them suitable materials for thermoelectric applications. In addition, the results of tight-binding (Hückel) model were in impressive agreement with transmission coefficient calculations obtained from Green's function method. Hence, I believe that these findings will strongly help developing fast and trustworthy the design of molecular electronics and thermoelectric materials.

## Conflicts of interest

The author declares no competing financial interest.

## Supplementary Material

RA-014-D4RA01463D-s001
