# Supplementary material for: Theoretical and modelling investigation of pendant groups effect on quantum interference for single-molecule junctions
Source: RSC Adv. 2024 May 7;14(21):14704–15. doi: 10.1039/d4ra01463d (PMC11075623; doi:10.1039/d4ra01463d)
Supplement: RA-014-D4RA01463D-s001 [file RA-014-D4RA01463D-s001.pdf]

Electronic Supplementary Material (ESI) for RSC Advances.
This journal is © The Royal Society of Chemistry 2024

Supplementary Information

Theoretical and modelling investigation of pendant groups effect on quantum interference for single-molecule junctions

Oday A. Al-Owaedi[ab*]

[a]Department of Laser Physics, College of Science for Women, University of Babylon, Hilla 51001, Iraq.
[b]Al-Zahrawi University College, Karbala, Najaf-Karbala Street, 56001, Iraq.

*Email: oday.alowaedi@uobabylon.edu.iq

Introduction

Studies of the electrical conductance of single molecules attached to metallic electrodes not only probe the fundamentals of quantum transport but also provide the knowledge needed to develop future molecular-scale devices and functioning circuits. [1-6] Owing to their small size (on the scale of Angstroms) and the large energy gaps (on the scale of eV), transport through single molecules can remain phase coherent even at room temperature, and constructive or destructive quantum interference (QI) can be utilized to manipulate their room temperature electrical [4-7] and thermoelectrical [8,9] properties. In this work, a combination of density functional theory (DFT) [10,11] methods, a tight binding (Hückel) [12] model (TBHM) and quantum transport theory (QTT) [13-22] have been utilized to inspect the DQI and its influence on electronic and thermoelectric properties of para- and meta-substituted oligo(phenylene-ethynylene) molecules with different central parts (carbonyl, diphenyl, ethane, Ethynylferrocene groups).

Theoretical Methods

All calculations in this work were carried out by the implementation of DFT in the SIESTA [23] code. It is used to obtain the optimized geometries of the structures, as shown in Figure S2. SIESTA software is an acronym derived from the Spanish Initiative for Electronic Simulations with Thousands of Atoms. The quantum transport theory (QTT) implemented in GOLLUM [24] software which is a program that computes the charge, spin and electronic contribution to the thermal transport properties of multi-terminal junctions has been utilized to calculate the electronic and thermoelectric properties of all molecular junctions in this work. All, theories and computational methods and procedures are shown in Figure S1.
The optimized geometries, ground state Hamiltonian and overlap matrix elements of each structure were self-consistently obtained using the SIESTA implementation of density functional theory (DFT). SIESTA employs norm-conserving pseudo-potentials to account for the core electrons and linear combinations of atomic orbitals to construct the valence states. The generalized gradient approximation (GGA) of the exchange and correlation functional is used with a double-ζ polarized (DZP) basis set, a real-space grid defined with an equivalent energy cut-off of 250 Ry. The geometry optimization for each structure is performed to the forces smaller than 20 meV/Å. The mean-field Hamiltonian obtained from the converged DFT calculation was combined with GOLLUM. The transmission coefficient $T(E)$ for electrons of energy E (passing from the source over molecule to the drain) is calculated via the relation:

$$T(E) = T_r\left\{\Gamma_R(E)G^R(E)\Gamma_L(E)G^{R\dagger}(E)\right\} \qquad (1)$$

In this expression,

$$\Gamma_{L,R}(E) = i\left(\Sigma_{L,R}(E) - \Sigma_{L,R}^{\dagger}(E)\right) \qquad (2)$$

$\Gamma_{L,R}$ describes the level broadening due to the coupling between left (L) and right (R) electrodes and the central scattering region, $\Sigma_{L,R}(E)$ are the retarded self-energies associated with this coupling.

$$G^R = \left(E\chi - H - \Sigma_L - \Sigma_R\right)^{-1} \qquad (3)$$

G^R is the retarded Green's function, where H is the Hamiltonian and χ is the overlap matrix (both of them are obtained from SIESTA). The transport properties is then calculated using the Landauer formula:

$$G = G_{\circ}\int dE\, T(E)(-\partial f(E,T)/\partial E) \quad (4)$$

where $G_0 = 2e^2/h$ is the conductance quantum, *e* is electron charge and *h* is the Planck's constant.

$$f(E) = \left(1 + exp\left((E - E_F)/k_BT\right)\right)^{-1} \quad (5)$$

where *f* is the Fermi-Dirac distribution function, T is the temperature and $k_B = 8.6\times10^{-5}$ eV/K is Boltzmann's constant [78].
DFT can give inaccurate value for the Fermi energy that the calculated conductance are obtained for a range of Fermi energies [25]. The thermopower or Seebeck coefficient *S* is defined as the difference of electrochemical potential per unit temperature difference developing across an electrically isolated sample exposed to a temperature gradient. The Seebeck coefficients and power factors is also informative. Provided the transmission function, *T*(*E*), can be approximated by a straight line on the scale of K_{BT}, the Seebeck coefficient is given by:

$$S \approx -L|e|T\left(\frac{dln\, T(E)}{dE}\right)_{E = E_F} \quad (6)$$

where *L* is the Lorenz number $L = \left(\frac{k_B}{e}\right)^2\frac{\pi^2}{3} = 2.44 \times 10^{-8}$ WΩK^{-2}. In other words, *S* is proportional to the negative of the slope of lnT(*E*), evaluated at the Fermi energy.
The power factor is the ratio of the real power absorbed by the load to the apparent power flowing in the circuit. Real power is the average of the instantaneous product of voltage and current and represents the capacity of the electricity for performing work. From the Seebeck coefficient, the power factor was calculated as given in equation (7)

$$P = GS^2 T \quad (7)$$

where *T* is the temperature *T* = 300 K, *G* is the electrical conductance and *S* is the thermopower.
In conventional devices the maximum efficiency of either heat transfer or current generation is proportional to the dimensionless thermoelectric figure of merit. The common measure for thermoelectric efficiency is given by the figure of merit, which is given by: [25]

$$ZT = \frac{GS^2}{k_{el} + k_{ph}}T \quad (8)$$

where *G* is the electrical conductance, S is Seebeck coefficient, k_{el} is the electron thermal conductance, $k_{ph}$is the phonon thermal conductance. The figure of merit is determined from the thermoelectric transport coefficients in equations 6, 9-10, and 12 in the linear response regime. [26-28]

$$G = \frac{2e^2}{h}k_0 \quad (9)$$

$$k_{el} = \frac{2}{hT}\left(K_2 - \frac{K_1^2}{K_0}\right) \quad (10)$$

In the expressions *e* = |*e*| is the absolute value of the electron charge, *h* is the Planck constant, and $T = (T_L + T_R)/2$ is the average junction temperature. The coefficients in 9 and 10 are defined as:

$$k_n = \int dE\, T_{el}(E)\left(-\frac{\partial f(E)}{\partial E}\right)(E - \mu)^n \quad (11)$$

where $T_{ph}(E)$ is the electron transmission, and the chemical potential $\mu \approx E_F$ is approximately given by the Fermi energy E_F of the Au electrodes.
The corresponding thermal conductance due to the phonons is given in linear response by:

$$k_{ph} = \frac{1}{h}\int_0^{\infty} dE\, ET_{ph}(E)\frac{\partial n(E,T)}{\partial T} \quad (12)$$

where $T_{ph}(E)$ is the phonon transmission and $n(E,T) = \{\exp(E/_{k_B}T) - 1\}^{-1}$ is the Bose function, characterizing the phonon reservoirs in the left and right electrodes.

Hence, an upper bound for ZT in the limit of vanishing phonon thermal transport $\kappa_{ph} \rightarrow 0$ is given by the purely electronic contribution [26] as

$$Z_{el}T = \frac{S^2 G}{k_{el}} T = \frac{S^2}{L} \quad (13)$$

Hence, the Lorenz number is $L = \kappa_{el}/GT$. With $Z_{el}T$, and depending on above the figure of merit is presented in a slightly different form as:

$$ZT = \frac{Z_{el}T}{1 + {k_{ph}}/{k_{el}}} \quad (14)$$

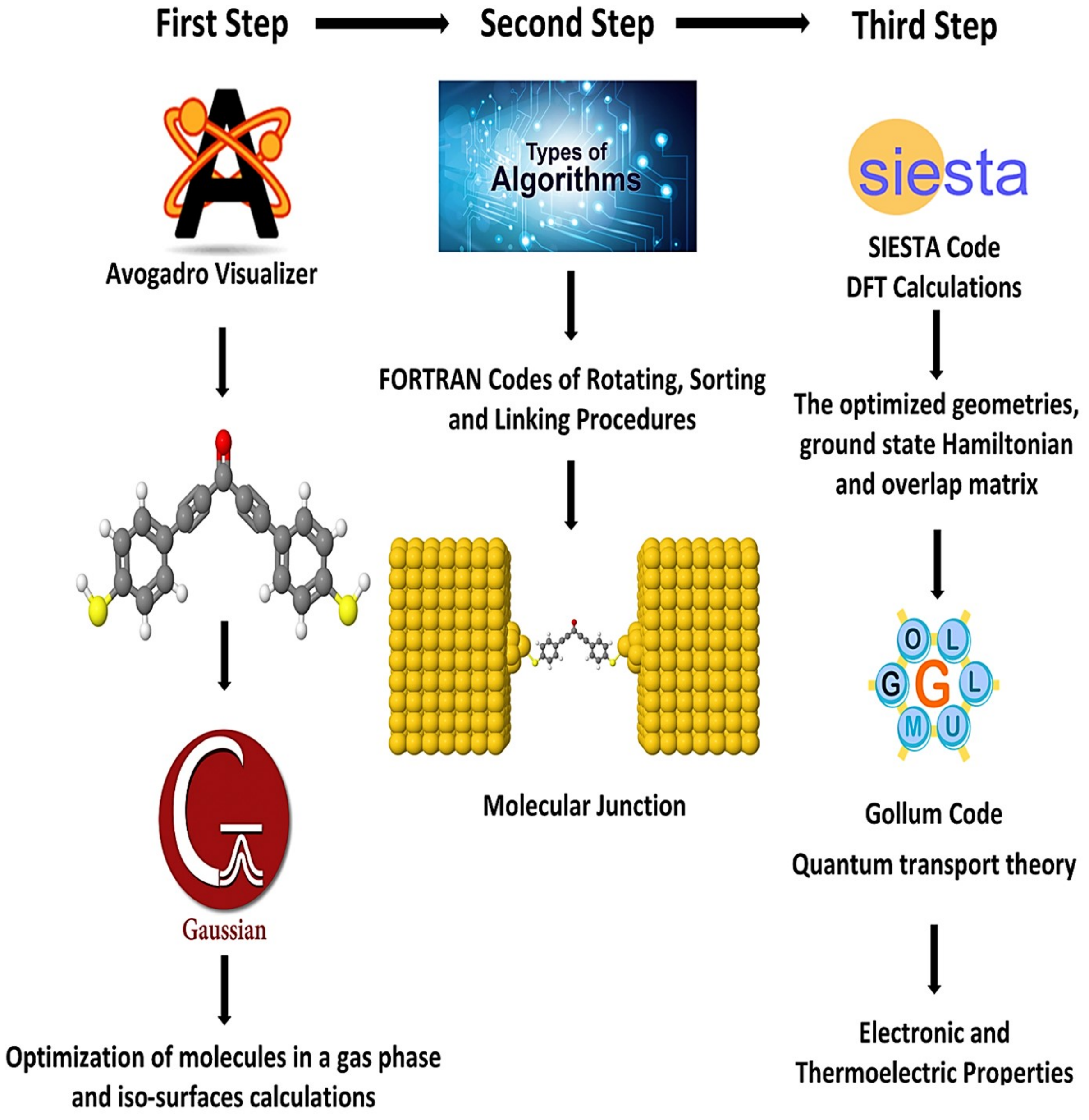

Figure S1. Computational methods and steps.

All molecules in a gas phase has been designed using Avogadro [29] visualizer, then the ground-state energy optimization of molecules and iso-surfaces calculations achieved using Gaussian [30] software at the B3LYP level of theory [31] with 6-31G** basis

set. [32,33] B3LYP is one of the most accurate and popular DFT functionals, and gives good results.[31-33] It is a so-called hybrid functional and is usually expressed in the following form:

$$F^{B3LYP}_{XC} = 1 - aF^{Slater}_{X} + aF^{HF}_{X} + bF^{B88}_{X} + cF^{LYP}_{C} + 1 - cF^{VWN}_{C} \quad (15)$$

where F_X^{Slater} refers to the Slater exchange, F_X^{HF} is the Hartree–Fock exchange, F_X^{88} is the Becke's exchange functional. F_C^{LYP} is the correlation functional of Lee, Yang, and Parr, and F_C^{VWN} is the correlation functional of Vosko, Wilk, and Nusair. The coefficients are a = 0.20, b = 0.72, and c = 0.81, which were adapted from another hybrid functional, B3PW91. The values of the coefficients were originally determined empirically by a linear least-squares fit to 116 experimentally determined energies. The second step involves the rotation, sorting and linking the molecules to the gold electrodes to obtain the theoretical models of molecular junctions (see Figure S3), using a set of FORTRAN algorithms. After that the molecular junctions have been optimized using SIESTA. [23] The data (Hamiltonian and overlap matrix) was then fed to the Gollum [24] code, which calculating the electronic and thermoelectric properties of all molecular junctions.

Theoretical Models of Molecular Junctions

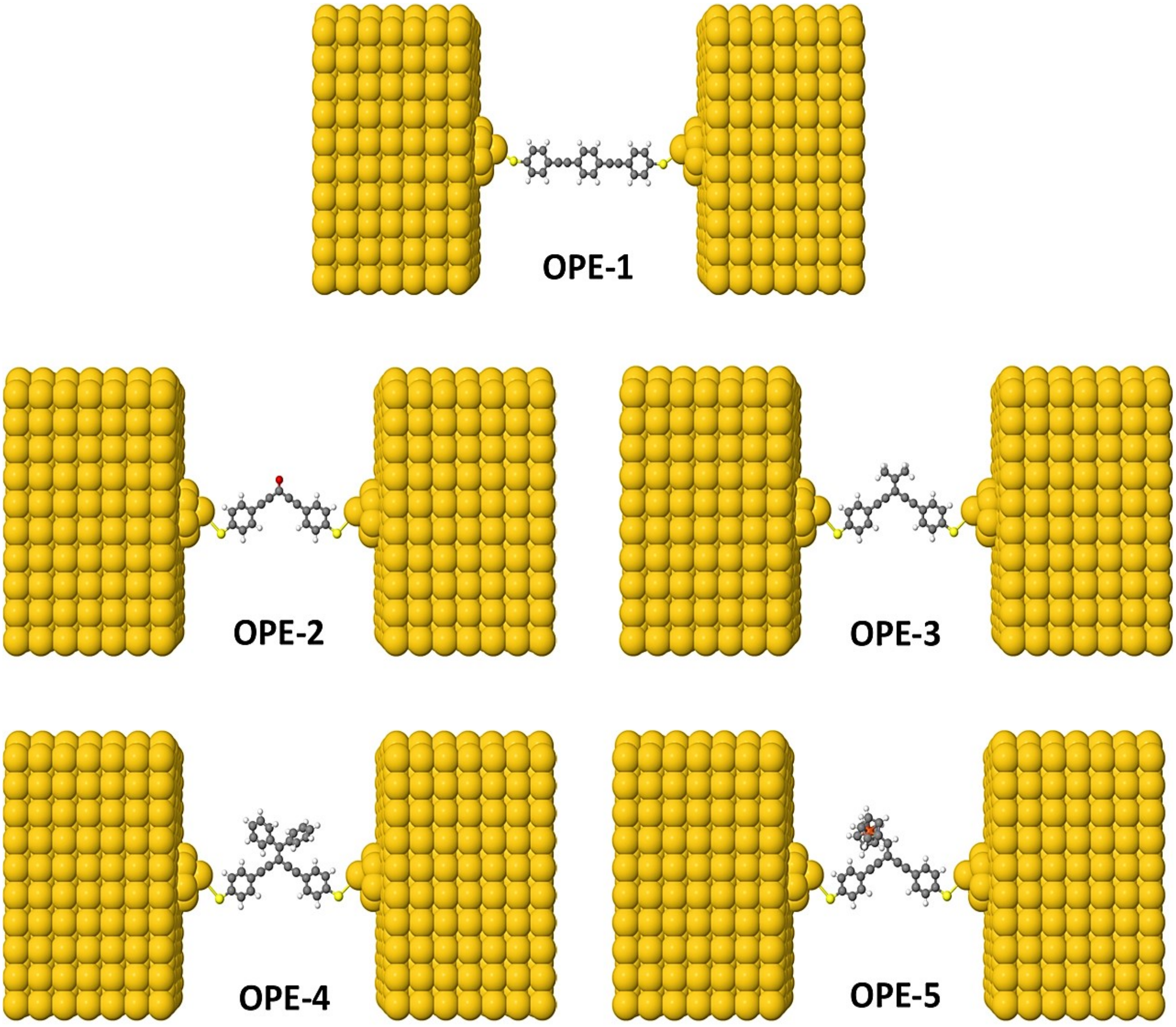

Figure S2. Theoretical models of all molecular junctions.

The theoretical models of all molecular configurations consist of optimized molecules attached two (111)-directed gold electrodes involving small 6-atom pyramidal gold leads, and each electrode constructed of eight layers of (111)-oriented bulk gold with each layer consisting of 6 × 6 atoms, and a layer spacing of 0.235 nm were employed to create the molecular junctions. These layers were then further repeated to yield infinitely long current carrying gold electrodes. From these model junctions the electronic and thermoelectric properties were calculated using the GOLLUM code.

Tight Binding Hückel Model

The neglect of tight-binding Hückel model (TBHM) of the interactions between electrons is considered a major defect, but it remains one of the widely used methods to visualize and understand the electronic properties of molecular junctions. [34]
One of the drawbacks of this kind of computational methods is the produced energy levels are diminished by a few eV in comparison with the accurate values relative to a vacuum, but the energy variances are usually appropriate to compare with DFT calculations. Anyway, this way is considered a powerful tool to award reasonable and precise results that could figure out the fundamental physics and resolve the problems.
In seeking to understand the transport behaviour of molecules and the relative effect of different central parts, a minimal tight-binding (Hückel) model (TBHM) has been constructed, as shown in Figure S3. The simplest tight-binding Hamiltonian of the parent is obtained by assigning a site energy ε to each diagonal and a nearest neighbor hopping integral γ between neighbouring sites, i.e., $H_{ii} = \varepsilon$ and $H_{ij} = \gamma$ if i, j are nearest neighbours. A minimal model of the central-substituted "daughters" (carbonyl, diphenyl, ethane, ethynylferrocene) is then obtained, as shown in Figure S3.

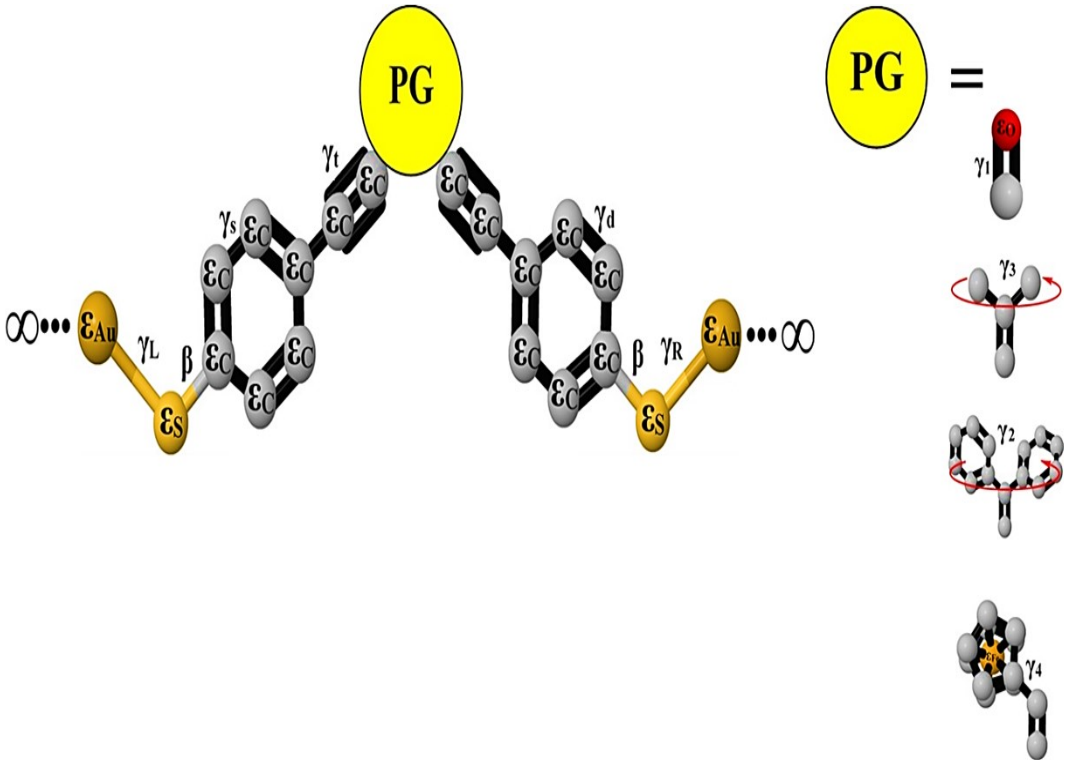

Figure S3. A minimal tight-binding (Hückel) representation of all molecules with different pandent groups. γ is the coupling elements, and ε is an onsite energy. Grey balls indicates to the onsite energy of carbon atoms (ε_C), light yellow balls refers to the onsite energy of sulfur atoms (ε_S), dark yellow balls is the onsite energy of gold atoms (ε_{Au}). Red balls indicates to the onsite energy of oxygen atoms (ε_O), Orange balls refers to the onsite energy of iron atoms (ε_{Fe}) and PG refers to pendant groups. The coupling element between carbon-carbon single bond is γ_S. γ_d is the coupling element between carbon-carbon double bonds. γ_t is the coupling element between carbon-carbon triple bonds. γ_L and γ_R are the left and right coupling elements between anchor groups and gold electrodes. The coupling element between carbon-oxygen double bonds is γ_1. γ_2

is the coupling element of the dihedral angle between phenyl rings. The coupling element of molecular rotation of CH3 fragments is γ_3. γ_4 is the coupling element of twisting angle.

Figure S3 shows a minimal tight-binding (Hückel) representation of m-OPE models with different pandent groups (carbonyl, diphenyl, ethyl, ethynyl ferrocene groups). The model involves a system connected to two one-dimension electrodes on both sides by weak nearest neighbor couplings γ_R and γ_L.

Figure S4 shows the transmission coefficints of OPE-2 model with carbonyl group as a central part of the molecule in three different model cases (I,II,III). The double bonds in carbonyl group are very reactivity. This reactive due to C=O electronegativity attributed to the oxygen atom and its two lone pairs of electrons. [35,36] One pair of the oxygen lone pairs are located in 2s orbital, while the other pair are in 2p orbital, where its axis is directed perpendicular to the direction of the π orbitals, as shown in Fig.ure S5. The double bond length of a carbonyl group is about 1.2 A° and the strength is about 176-179 kcal/mol. [35] The Carbonyl group properties are directly tied to its electronic structure as well as geometric positioning. It is possible to correlate the length of a carbonyl bond with its polarity; the longer the bond meaing the lower the polarity.

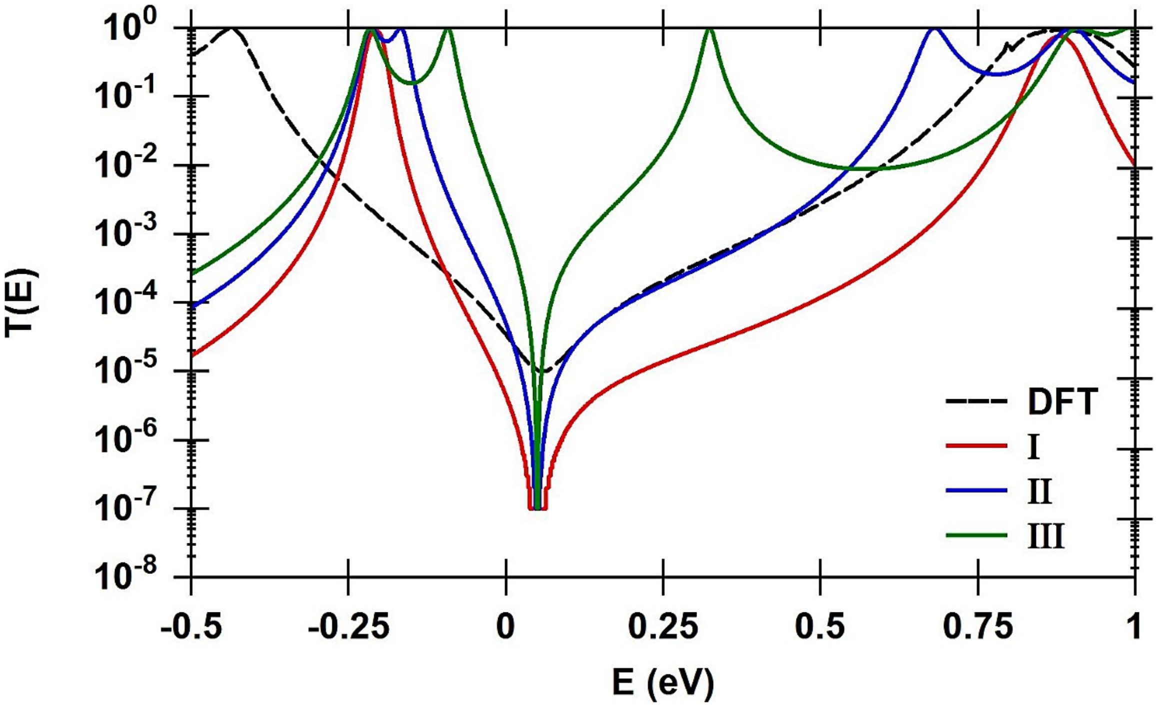

Figure S4. Transmission coefficients of OPE-2 molecule in three different model cases (I,II,III), as a function of electrons energy. The dashed black curve is the DFT-transmission coefficient.

In this context, TBHM of this work visualizes the double bond length as $\boldsymbol{\gamma_1}$, which has been changed from 1.5 A° ($\boldsymbol{\gamma_1}$= 2) for the case I to 1.7 A° ($\boldsymbol{\gamma_1}$= 1) for case II and 1.9 A° (γ_1= 0.5) for case III. All other parameters β= 1, γ= 0.5, ε_C= 0.1, ε_S= 2.5, ε_{Au}= 0.5, $\gamma_S = 0.3$, $\gamma_d = 0.5$, $\gamma t = 0.7$, $\gamma_L = \gamma_R$ = 0.2, are fixed for all cases. The changing of the polar double bonds ($\boldsymbol{\gamma_1}$) value of this structure, manipulating the electrical dipole moment between negative and positive charges of oxygen and carbon atoms respectively, which leading to different transport paths of de Broglie waves, and consequently to DQI. An exsellent agreement with DFT is obtained for case II, as shown in Figure S4.

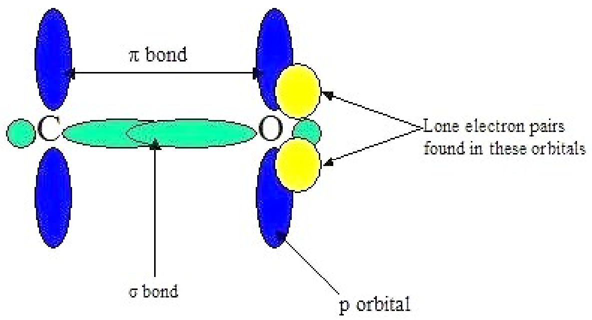

Figure S5. Chemical structure of oxgen-carbon double bonds. [35]

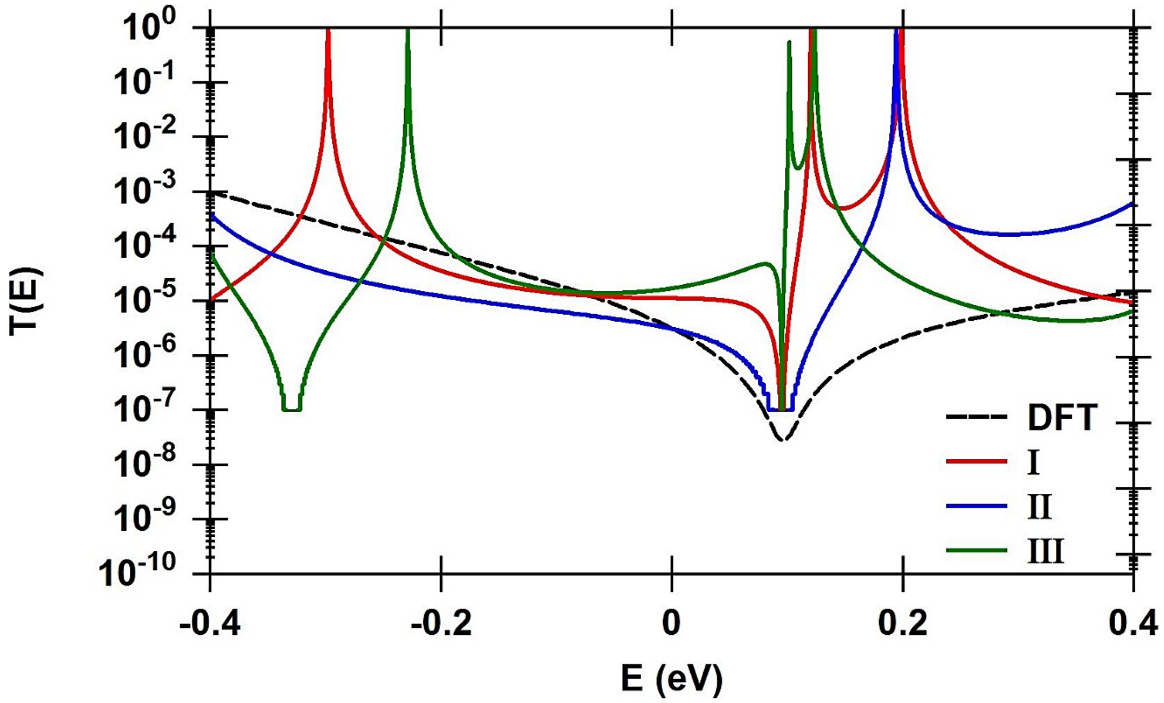

Figure S6. Transmission coefficients of OPE-3 molecule in three different model cases (I,II,III), as a function of electrons energy. The dashed black curve is the DFT-transmission coefficient.

The CH3 fragments of ethane substitute are joined by a carbon-carbon sigma bonds allowing them to rotate about these bonds. In terms of a Newman projection [37], the free rotation around single bonds, there will be various conformations for ethane compound. Up to six unique conformations may be drawn, and each conformation is drawn by rotation of either the proximal or distal atom 60°. Of these six conformations, three will be in a staggered conformation, while the other three will be in an eclipsed conformation. These six conformations can be represented in a relative energy diagram, as shown in Figure S7. Staggered conformations are the lower energy arrangements. This is because each side group is 60° apart from the others, so there is no torsional strain; the side groups of the front carbon are maximally spread out from those of the back carbon, which decreases the possibility of interaction. Eclipsed conformations are higher in energy than staggered due to bond straining. In this arrangement, the side groups of the front carbon are directly in front of the side groups of the back carbon; so when looking from the side you would only see the side groups of the closer carbon. [38]

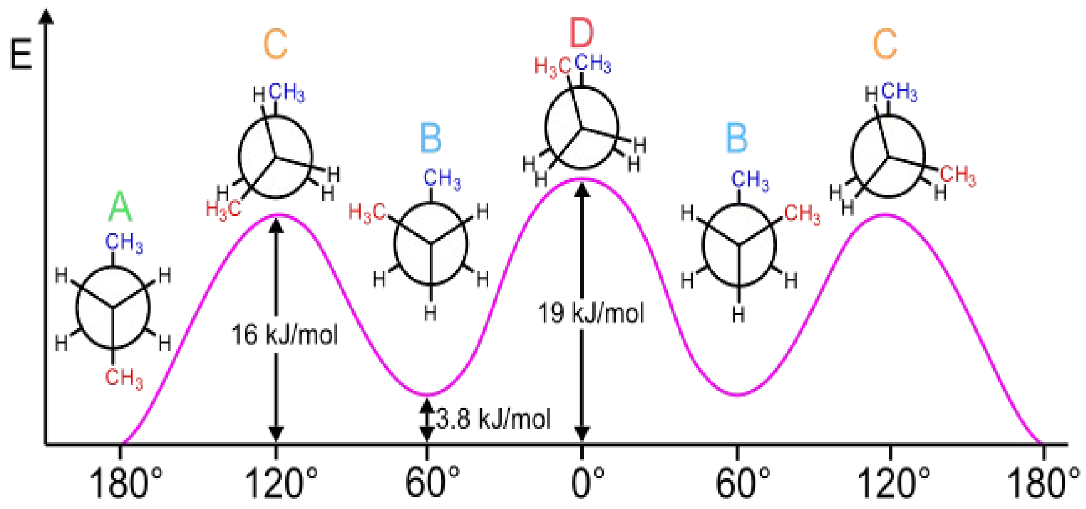

Figure S7. Sketch shows the energy diagram of the six unique conformations of methyl fragments of the ethane substitute.[37]

Here, Hückel model of this work depicts the molecular rotation by changing the value of γ_3 to obtain different eclipsed conformations, and different DQIs. Figure S6 illustres *T*(*E*) as a function of electrons energy of OPE-3 model in three cases. For case I, $\gamma_3 = 0.4$; case II, $\gamma_3 = 0.7$, and for case III, $\gamma_3 = 0.3$. The perfect consistency between DFT and TBHM for case II, as shown in Figure S6.

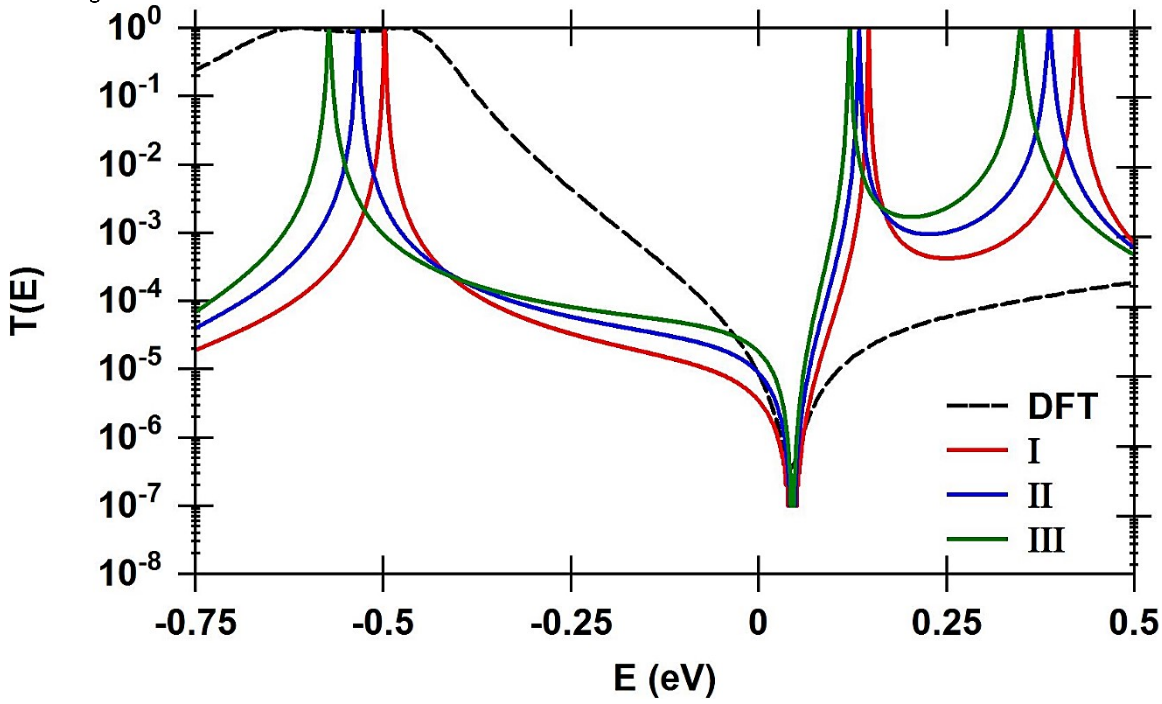

Figure S8. Transmission coefficients of OPE-4 molecule in three different model cases (I,II,III), as a function of electrons energy. The dashed black curve is the DFT-transmission coefficient.

Figure S8 shows the transmission coefficints of OPE-4 model with Y-shaped enediyne (diphenyl group) as a central part of the molecule in three different model cases (I,II,III). A diphenyl compound involves two phenyl rings linked to carbon atom by single carbo-carbon bonds. The aromaticity of diphenyl is distinguished by the π-conjugation property.[37,39] It is conformational enantiomorphism as shown in Figure S9, since diphenyl itself is not planar, one phenyl ring being slightly twisted or canted in relation to the other as a consequence of steric crowding. The resulting chiral conformation, having a dihedral angle of about 45°, equilibrates rapidly with its enantiomer by rotation about the connecting single bond. Note that a conformation having a 90° dihedral angle is achiral, as a consequence of a plane of symmetry.

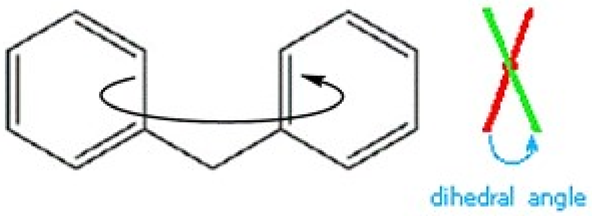

Figure S9. Sketch shows the conformations of a diphenyl group. [38]

Here TBHM considers the dihedral angle between phenyl rings and visualize it as $\boldsymbol{\gamma_2}$, as shown in Figure 3S. The manipulating with $\boldsymbol{\gamma_2}$ deceasing the π-conjugation degree, and hence producing DQIs. Figure S10 shows the transmission coefficient of model OPE-4. All parameters β= 1, γ= 0.5, ε_C= 0.1, ε_S= 2.5, ε_{Au}= 0.5, $\gamma_S = 0.3$, $\gamma_d = 0.5$, $\gamma t = 0.7$, $\gamma_L = \gamma_R$ = 0.2, are fixed for all cases (I,II,III). For case I, $\boldsymbol{\gamma_2}$ = 0.7; case II, $\boldsymbol{\gamma_2}$ = 0.9, and for case III, $\boldsymbol{\gamma_2}$ = 1.1. Herein, Hückel's model visualization of the twist angle ($\boldsymbol{\gamma_2}$) in II case consistents with DFT calculations.

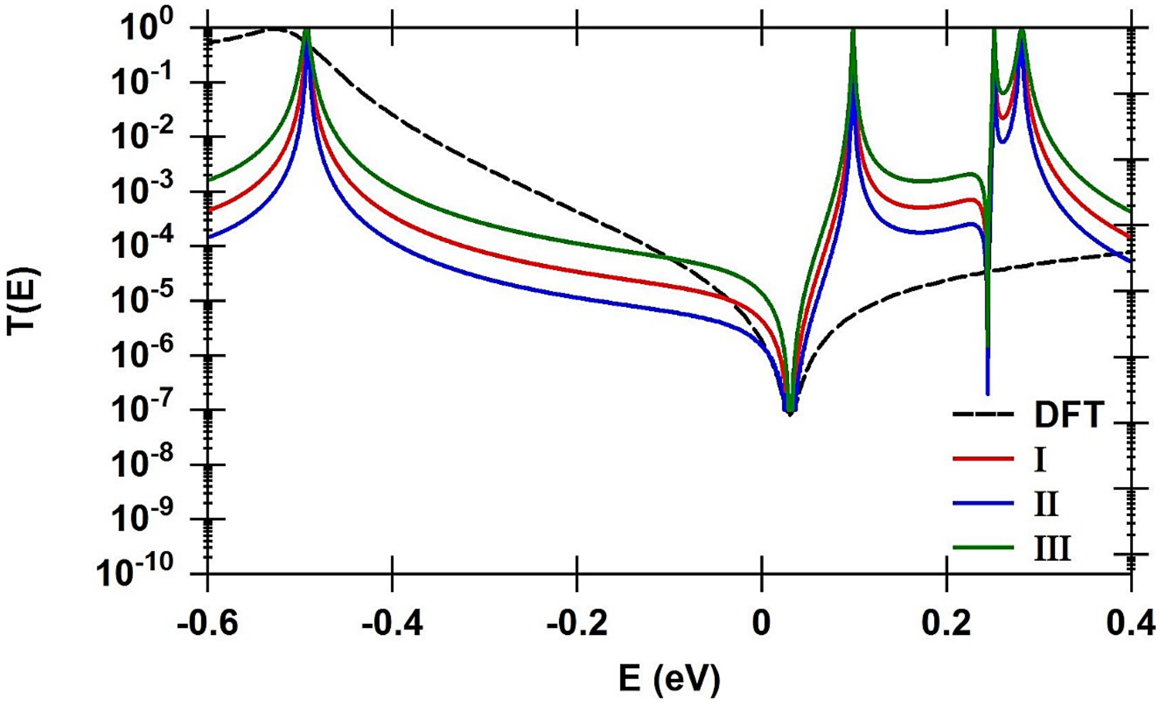

Figure S10. Transmission coefficients of OPE-5 molecule in three different model cases (I,II,III), as a function of electrons energy. The dashed black curve is the DFT-transmission coefficient.

The OPE-5 Model contains an ethynylferrocene group, which possess an iron metal accommodated between two rings of cyclopentadienyl (CP), as shown in Figure S3. Ferrocene is an example of a broader class of 3D metallocenes which posses high chemical and thermal stability. [40] It consists of two cyclopentadienyl (Cp) rings bound by a central iron atom, which may rotate about the Cp–Fe–Cp axis. In the condensed phase the ground state is found to be the staggered configuration, while in the gas phase it corresponds to the eclipsed configuration. For the isolated molecule the difference between the staggered and the eclipsed configurations is about ≈ 20 meV. [40]

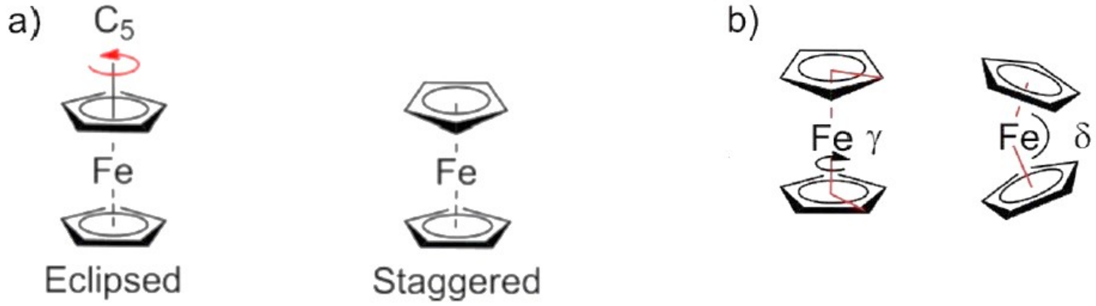

Figure S11. Sketch shows **a**) Eclipsed and staggered configurations of ferrocene compound; **b**) the twist and bend angles (γ and δ respectively) [39].

The conformational interchange process from eclipsed to staggered involves a twist around the C5 rotation axis that changes the torsion angle γ connecting any carbon atom of one ring to the corresponding carbon atom of the second ring through the two ring centroids, as shown in Figure S11. Figure S10 exhibites the transmission coefficient of OPE-5 with ethynylferrocene group as a cenral part. Hückel model visualizes the the twist angle as $\boldsymbol{\gamma_4}$. For case I, $\boldsymbol{\gamma_4}$ = 0.3; case II, $\boldsymbol{\gamma_4}$ = 0.1, and for case III, $\boldsymbol{\gamma_4}$ = 0.5. The consent between TBHM and DFT in case II.

References

1 J. Chen, M. A. Reed, A. M. Rawlett, J. M. Tour, *Science*, 1999, **286**, 1550-1552.
2 M. A. Reed, C. Zhou, C. J. Muller, T. P. Burgin, J. M. Tour, *Science*, 1997, **278**, 252-254.
3 B. Q. Xu, N. J. Tao, *Science*, 2003, **301**, 1221-1223.
4 C. Jia, X. Guo, *Chem. Soc. Rev.*, 2013, **42**, 5642-5660.
5 L. Venkataraman, J. E. Klare, C. Nuckolls, M. S. Hybertsen, M. L. Steigerwald, *Nature*, 2006, **442**, 904-907.
6 H. Ozawa, M. Baghernejad, O. A. Al-Owaedi, V. Kaliginedi, T. Nagashima, J. Ferrer, T. Wandlowski, V. M. García-Suárez, P. Broekmann, C. J. Lambert, M. Haga, *Chem. Eur. J.* 2016, **22**, 12732-12740.
7 D. Stefani, K. J. Weiland, M. Skripnik, C. Hsu, M. L. Perrin, M. Mayor, F. Pauly, H. S. J. van der Zant, *Nano Lett.*, 2018, **18**, 5981–5988.
8 J. C. A. Eichler, J. M. G. Ceballos, R. R. A. Bachtold, *Nat. Nanotech.*, 2012, **7**, 301-304.
105 C. J. Lambert, *Chem. Soc. Rev.*, 2015, **44**, 875-888.
9 C. M. Finch, V. M. Garcia-Suarez, C. J. Lambert, *Phys. Rev. B.*, 2009, **79**, 033405.
10 J. P. Perdew, K. Burke, M. Ernzerhof, *Phys. Rev. Lett.*, 1996, **77**, 3865.
11 P. J. Stephens, F. J. Devlin, C. F. Chabalowski, M. J. Frisch, *J. Phys. Chem.*, 1994, **98**, 11623–11627.
12 E. Estrada, *Proc. R. Soc. A.*, 2017, **474**, 0721.
13 J. P. Perdew, Y. Wang, *Phys. Rev. B.*, 1992, **45**, 13244.
14 J. Ferrer, C. J. Lambert, V. M. García-Suárez, D. Zs Manrique, D. Visontai, L. Oroszlany, R. Rodríguez-Ferradás, I. Grace, S. W. D. Bailey, K. Gillemot, H. Sadeghi, L. A. Algharagholy, *New J. Phys.*, 2014, **16**, 93029.
15 M. Jirásek, H. L. Anderson, M. D. Peeks, *Acc. Chem. Res.*, 2021, **54**, 3241–3251.
16 C. J. Judd, A. S. Nizovtsev, R. Plougmann, D. V. Kondratuk, H. L. Anderson, E. Besley, A. Saywell, *Rev. Lett.*, 2020, **125**, 206803.
17 Z. Chen, J. Deng, S. Hou, X. Bian, J. L. Swett, Q. Wu, J. Baugh, L. Bogani, G. A. D. Briggs, J. A. Mol, C. J. Lambert, H. L. Anderson J. O. Thomas, *J. Am. Chem. Soc.*, 2023, **145**, 15265–15274.
18 S. Richert, J. Cremers, I. Kuprov, M. D. Peeks, H. L. Anderson, C. R. Timmel, *Nat. Commun.*, 2017, **8**, 14842.
19 B. Limburg, J. O. Thomas, G. Holloway, H. Sadeghi, S. Sangtarash, J. Cremers, A. Narita, K. Müllen, C. J. Lambert, G. D. Andrew, B. J. Mol, H. L. Anderson, *Adv. Func. Mat.*, 2018, **28**, 1803629.
20 B. Limburg, J. O. Thomas, G. Holloway, H. Sadeghi, S. Sangtarash, J. Cremers, A. Narita, K. Müllen, C. J. Lambert, G. D. Andrew, B. J. Mol, H. L. Anderson, *Adv. Func. Mat.*, 2018, **28**, 1803629.
21 G. Sedghi, L. J. Esdaile, H. L. Anderson, V. M. García-Suárez, C. J. Lambert, S. Martin, D. Bethell, S. J. Higgins, R. J. Nichols, *Nature Nanotech.*, 2011, **6**, 517-523.
22 N. Algethami, H. Sadeghi, S. Sangtarash, C. J. Lambert, *Nano Lett.*, 2018, **18**, 4482-4486.
23 E. Leary, B. Limburg, S. Sangtarash, A. Alanazy, I. Grace, K. Swada, L. J. Esdaile, M. M. Noori, T. González, G. Rubio-Bollinger, H. Sadeghi, N. Agrait, A. Hodgson, S. J. Higgins, C. J. Lambert, H. L. Anderson, R. Nichols, *J. Am. Chem. Soc.*, 2018, **140**, 12877–12883.
24 C. J. Lambert, *Chem. Soc. Rev.*, 2015, **44**, 875-888.
25 U. Sivan, Y. Imry, *Phys. Rev. B.*, 1986, **33**, 551.
26 K. Esfarjani, M. Zebarjadi, Y. Kawazoe, *Phys. Rev. B.*, 2006, **73**, 085406.
27 K. Müller, *J. Chem. Phys.*, 2008, **129**, 044708.
28 M. D. Hanwell, D. E. Curtis, D. C. Lonie, T. Vandermeersch, E. Zurek, G. R. Hutchison, *J. Cheminform.*, 2012, **4**, 1-17.
29 H. B. J. Schlegel, S. Binkley, J. A. Pople, *J. Chem. Phys.*, 1984, **80**, 1976-1981.
30 A. D. Becke, *J. Chem. Phys.*, 1993, **98**, 5648–5652.
31 G. A. Petersson, A. Bennett, T. G. Tensfeldt, M. A. Al-Laham, W. A. Shirley, J. Mantzaris, *J. Chem. Phys.*, 1988, **89**, 2193–2198.
32 G. A. Petersson, M. A. Al-Laham, *J. Chem. Phys.*, 1991, **94**, 6081–6090.

33 K. Walczak, L. S. Edward, *Centr. Eur. J. Phys.*, 2005, **3**, 555-563.
34 S. Patai, J. Zabicky, London-New York-Sydney, *Interscience*, 1966, **1**.
35 R. H. Mitchell, V. S. Iyer, *J. Am. Chem. Soc.*, 1996, **118**, 2903-2906.
36 R. J. Davidson, D. C. Milan, O. A. Al-Owaedi, A. K. Ismael, R. J. Nichols, S. J. Higgins, C. J. Lambert, D. S. Yufita, A. Beeby, *RSC Adv.*, 2018, **8**, 23585–23590.
37 M. S. Newman, *J. Chem. Educ.*, 1955, **32**, 344.
38 C. Engtrakul, L. R. Sita, *Nano Lett.*, 2001, **1**, 541.
39 O. A. Al-Owaedi, S. Bock, D. C. Milan, M. Oerthel, M. S. Inkpen, D. S. Yufit, A. N. Sobolev, N. J. Long, T. Albrecht, S. J. Higgins, M. R. Bryce, R. J. Nichols, C. J. Lambert, P. J. Low, *Nanoscale*, 2017, **9**, 9902–9912.
40 A. W. Kaspi-Kaneti, I. Tuvi-Arad, *Organometallics*, 2018, **37**, 3314-3321.
